# Granulocyte colony-stimulating factor gene rs1042658 variant and susceptibility to idiopathic recurrent pregnancy loss: A case-control study

**Published:** 2018-01

**Authors:** Mahboobeh Nasiri, Kobra Jahangirizadeh

**Affiliations:** *Department of Biology, Islamic Azad University, Arsanjan Branch, Arsanjan, Iran.*

**Keywords:** Granulocyte colony-stimulating factor, Recurrent pregnancy loss, Polymorphism

## Abstract

**Background:**

Granulocyte colony-in stimulating factor (*G-CSF*) gene can be a potential candidate gene implicated recurrent pregnancy loss (RPL), a common complication of pregnancy with the prevalence of 1-5% among women of reproductive age.

**Objective:**

To investigate the association between rs1042658 polymorphism in the 3' untranslated region (3'UTR) of *G-CSF* gene and the risk of unexplained RPL among Iranian women.

**Materials and Methods:**

In total, 122 women with unexplained RPL and 140 healthy postmenopausal women as a control group were enrolled in this case-control study. Tetra-primer amplification refractory mutation system-polymerase chain reaction was performed to determine the rs1042658 genotypes in all subjects.

**Results:**

Statistically significant differences were detected between the distribution frequencies of both heterozygote CT, and carriage of T allele (TT+CT) genotypes of the rs1042658 between case and control groups. Allelic association was not observed with RPL.

**Conclusion:**

Regarding the results of the present study, *G-CSF* rs1042658 gene polymorphism could be considered as a probable risk factor for unexplained RPL among Iranian women.

## Introduction

Cytokines were proved to have a large impact on the transplantation tolerance, such as maintenance of semi-allograft fetus during pregnancy (1, 2). 

Granulocyte colony-stimulating factor (G-CSF or CSF3) is stimulated in response to inflammatory conditions and injuries (3). 

G-CSF is transducing the specific biological signals by binding to the cell surface receptor (G-CSFR) expressed mostly on hematopoietic cell lineages and some non-hematopoietic cells e.g. endothelial cells, placenta, trophoblastic cells, and granulosa luteinized cells (4). 

Among G-CSF biological actions, a cascade of tolerogenic events are more prominent composing of; the blockade of pro-inflammatory cytokine production, up-regulation of the anti-inflammatory cytokines e.g. interleukin (IL)-4 and IL-10, reducing interferon γ production in lymphocytes, as well as, shifting the T-helper (Th)-1/Th-2 balance toward Th-2 responses, and accumulation of regulatory T cells (5, 6).

Recurrent pregnancy loss (RPL) is a complex disease, the cause of which is unknown for more than 50% of the cases, called idiopathic RPL (7). Defects in the balance regulation of Th-1/Th-2/Th-17 and regulatory T cells (Treg) function has been implicated in the pathobiology of RPL (8, 9). 

The association of the G-CSF and several autoimmune diseases, such as rheumatoid arthritis and systemic lupus erythematosus was demonstrated in many studies, which both are Th-1/Th-17 diseases as RPL (10, 11). Those evidences encouraged us to assess a possible association between the *G-CSF* gene, mapped on chromosome 17q11.2-12, with RPL (12).

In this study, we investigated whether or not genotype and allele frequencies of the rs1042658 single nucleotide polymorphism (SNP) in the 3′ untranslated region of the *G-CSF *gene confer susceptibility to RPL in Iranian women.

## Materials and methods

The case-control study consisted of women with the history of at least two unexplained miscarriages, which were screened for polymorphism of the G-CSF gene. The presence of anatomical uterine abnormalities detected by hysteroscopy, positive cultures for chlamydia and mycoplasma, paternal and maternal chromosomal aberration, and endocrine disorders were considered as exclusion criteria. As one of the main objectives of the study was to identify different RPL-related factors, we, therefore, collected detailed information including abortion type, the number of live children, and oral contraceptive use from all participants. A total of 122 RPL women as the case group and 140 healthy controls were recruited into the study. Pregnant women were excluded from the study. 

Population-based controls were randomly sampled from postmenopausal women referred for an annual checkup to the Shafa Hospital Laboratory, Shiraz, Iran. They have never experienced any pregnancy complications even miscarriage but also have at least two live births.

**DNA extraction and rs1042658 genotyping**

Genomic DNA was extracted from the white blood cells of each sample using a standard salting out method (13). The quality of the extracted genomic DNA was evaluated on 1% agarose gel electrophoresis. The fast, simple, and cost-effective Tetra-primer amplification refractory mutation system-PCR (T-ARMS PCR) was used to determine different genotypes regarding the selected polymorphism using the following four primers: forward outer (FO), 5'-GATGAGCCG CTGTGAGCCCCTGGTCCTGAG-3'; reverse outer (RO), 5'-CAGACGAACTTGAGAACTTT CCTCCTCCTCCC-3'; forward inner (FI), 5'-G GTGCCTGGACATTTGCCTTGCTGTAC-3'; and reverse inner (RI), 5'-TGCTCCCTCCCA CATCCCCAGTCCACA-3'. The FO and RI primers were used to amplify T allele fragment (456bp), whereas FI and RO primers were used to amplify C allele fragment (335bp). FO and RO primers amplified a 738bp fragment as an internal control, which is genotype-independent amplicon. The reaction mixture was as follows: 6.25µl PCR master mix (Yekta Tajhiz Azma, Iran); 1µl FO primer (10µM); 1µl RO primer (10µM); 3µl FI (10µM); 3 µl RI primer (10µM), and 1.0µl DNA template. Double-distilled water was added to obtain a final reaction volume of 12.5µl. The reaction mixture was heated in a thermocycler (ABI, Perkin Elmer 9600) under the following condition: 95°C for 5min, 30 cycles (95°C for 1 min; 70°C for 1 min), and 72°C for 1 min, and final extension at 72°C for 7 min. PCR products were resolved on 2% agarose gel electrophoresis and the bands were detected under UV light using UVIsoft software (UVItec Cambridge, UK) following staining by DNA safe stain dye.

**Ethical consideration**

This study was approved by the Islamic Azad University, Arsanjan Branch Ethics Committee. Written informed consent was obtained from all participants.

**Statistical analysis**

Student’s t-test and Chi-square test were used to compare the continuous and nominal variables among RPL (cases) and control women, respectively. The X^2^ test was also used to test Hardy- Weinberg equilibrium (HWE). Allelic and genotyping association of the selected SNP were evaluated using logistic regression and calculation of odds ratio (OR) with 95% confidence interval (CI). Considering the significant age difference between RPL patients and controls, all logistic regression analysis was adjusted for age. In order to assess the risk of confounding factors on the RPL susceptibility, the data were subjected to regression analysis considering RPL as the dependent variable and each of the risk factors as independent factors. 

Statistical analysis was performed using the Statistical Package for Social Sciences (version 16; SPSS Inc., Chicago, IL). p value<0.05 was considered statistically significant.

## Results

**General characteristics**

The general characteristics of the study participants are shown in table I.

**Genotypes and allelic distribution of rs1042658**

Table II shows the genotypic and allelic distributions of the rs1042658 polymorphism in the cases and controls. The observed frequency of the genotypes in control group was in agreement with those expected based on Hardy-Weinberg equilibrium (X^2^=0.35; df=1; p=0.55).

The frequency of the heterozygote genotype CT was significantly higher in controls compared to cases (43% vs. 33%), then it showed a protective effect against RPL (OR: 0.42, 95%CI: 0.18-0.98, p=0.04). 

The frequency of the genotypes carried at least one T-allele was significantly higher in controls compared to cases and hence protect women from RPL (OR: 0.44, 95%CI: 0.2-0.99, p=0.04). Distribution of the T-allele was not significantly differed between cases and controls (OR: 0.89, 95%CI: 0.6- 1.31, p=0.55). Logistic regression analysis confirmed the association of the G-CSF rs1042658 gene variant, after adjusting for the RPL variables; age, oral contraceptive use and body mass index (Table III). 

**Table I:** Demographic and clinical features of cases and controls

**Characteristics**		**Case group (n= 122)**	**Control group (n= 140)**	**p-value ** *
Age (mean±SD)		35.5 ± 12.5	57.8 ± 6.8	<0.001
Abortion (mean±SD)		3.3 ± 1.2	0.0 ± 0.0	-
Abortions				
	2	22	0.0 ± 0.0	-
	≥3	100	0.0 ± 0.0	-
Abortion type				
	Primary	62 (51) †	0.0 ± 0.0	-
	Secondary	60 (49)	0.0 ± 0.0	-
Number of pregnancies		4.61 ± 2.34	5.36 ± 2.3	0.01
Number of children		1.3 ± 2.22	5.36 ± 2.3	0.001
Body mass index (BMI, kg/m^2^)		25.4 ± 2.9	26.9 ± 3.2	<0.001

* Student's *t*-test; p<0.05

† percentage of total within each group/ subgroup

**Table II:** Allelic and genotypic frequencies of *G-CSF* rs1042658 gene polymorphism

**polymorphism**		**Controls (n= 140)**	**Cases (n= 122)**	**p-value**	† **OR**	**95%CI**
Genotype						
Co-dominant model						
	CC	70 (50)	70 (57)	-	1	Reference
	CT	60 (43)	40 (33)	0.04	0.42	0.18-0.98
	TT	10 (7)	12 (10)	0.45	0.56	0.12-2.5
Dominant model (T)						
	TT+TC (T+)	70 (50)	52 (43.3)	0.04	0.44	0.2-0.99
Allele						
	C	200 (71)	180 (74)	-	1	Reference
	T	80 (29)	64 (26)	0.55	0.89	0.6-1.31

OR: Odd ratio;

CI: Confidence interval

p<0.05, Logistic regression

† Adjusted for age

**Table III:** Influence of rs1042658 variant on the risk of unexplained RPL after adjustment for potential variables

**Factors**	**p-value**	**OR**	**95%CI**
Age	< 0.001	0.83	0.80- 0.87
Oral contraceptive use	0.23	1.66	0.72- 2.8
BMI	0.004	0.82	0.72- 0.94
rs1042658 (T+)†	0.04	0.42	0.18- 0.96

BMI: Body mass index

Multivariate logistic regression

† Homozygote+ heterozygote carriers

**Figure 1:**
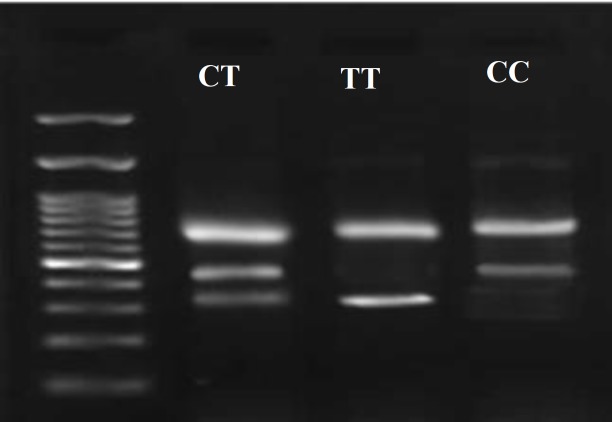
Schematic representation of the electrophoretic results of the T-ARMS PCR for rs1042658 variant. Heterozygote CT genotype is determined by the size fragments of 738 bp, 456 bp, and 335 bp. The TT homozygote is determined by 738 bp and 335 bp fragments. The CC homozygote is shown in the last line in right by fragments of 738 bp and 456bp fragment in length. The 100 bp size marker ladder is loaded in first line of the left corner

## Discussion

The key finding of the current study is that the rs1042658 genetic variant of *G-CSF* gene is associated with decreasing susceptibility to RPL in the dominant model for allele T, which this association is apparently independent of the confounding variables including age, oral contraceptive use, and body mass index. 

G-CSF cytokine is a recently discovered molecular marker with potent therapeutic property and pivotal roles in curtailing pregnancy failure (14, 15). G-CSF guarantees the health of the pregnancy by enhancing embryo implantation and ovarian function (4) and therefore, by increasing the endometrial thickness contributes to reduced pregnancy loss (16). Expression profiling of the cells followed by the administration of recombinant human G-CSF (rhG-CSF) resulted in the significant increase in the genes involved in cell migration and embryo implantation (Integrin alpha-V/beta-3 and Plasminogen Activator Urokinase Receptor), angiogenesis (Thymidine Phosphorylase), and cell proliferation control (CD40 and CD40 Ligand) (17), and also an increase in regulatory T (Treg) cells and dendritic cells (17, 18). 

The immunological factors are considered critical for embryo implantation, and from this point of view, it is proven that the G-CSF attenuated the production of pro-inflammatory cytokines including IL-1β, IL-12, interferon (IFN)-γ, IL-18, and tumor necrosis factor (TNF)-α, and in turn up-regulated anti-inflammatory cytokines, IL-4 and IL-10 (19, 20). 

Pregnancy is known as a Th-2 phenomenon, which high and constitutive expression of IL-4 and IL-10 anti-inflammatory cytokines guarantee the maintenance of tolerogenic environment during pregnancy (21). IL-17 producing T (Th-17) cells have a high pathogenic potential by inducing inflammation (22). Previous studies demonstrated that increased Th-17 and hence, an elevated level of IL-17 in pregnancy decidua might be disadvantageous for maintenance of pregnancy. G-CSF influences the IL-17-associated immune responses by negatively regulation of IL-17 production (23, 24). Besides these two hypotheses, the role of Treg cells in the maintenance of pregnancy has come into scene recently (25). Reduced number of both circulating and decidua Treg is an obvious marker in women with miscarriage (26). Administration of G-CSF increases the number of Treg in decidua in pregnant women and therefore, reduces the risk of pregnancy failure (27, 28). Further evidence strongly supported the crucial role of IL-10 in G-CSF-Treg interplay (29). 

We detected an association between RPL and variant in *G-CSF*, but the specific function of rs1042658T allele is unknown. We hypothesized that the substitution of the C allele with the T allele resulted in an increased expression of G-CSF by several processes including ribosome binding, initiation, and elongation of the translation. Consequently, the higher quantity of G-CSF up-regulates the expression of target immune modulation genes (like *IL-10*) and the increase the number of Treg during pregnancy which improves the implantation and protects the fetus to term.

## Conclusion

In conclusion, our results showed that the polymorphic T allele of the *G-CSF* rs1042658 polymorphism is associated with decreased risk of RPL among Iranian women, which this association is independent of the profounding risk factors of the RPL. 
